# Hemolytic uremic syndrome associated with *Escherichia coli* O157:H7 infection in older adults: a case report and review of the literature

**DOI:** 10.1186/s13256-016-0970-z

**Published:** 2016-06-15

**Authors:** Heidi Ko, Hossein Maymani, Cristhiam Rojas-Hernandez

**Affiliations:** 1 Department of Internal Medicine, The University of Texas Health Science Center, Houston, TX USA; 2 Division of Internal Medicine, Section of Medical Oncology and Hematology, The University of Texas MD Anderson Cancer Center, Houston, TX USA; 3 Division of Internal Medicine, Section of Benign Hematology, The University of Texas MD Anderson Cancer Center, Houston, TX USA

**Keywords:** Case report, Hemolysis, Microangiopathy, Elderly

## Abstract

**Background:**

Hemolytic uremic syndrome associated with Shiga toxin-producing *Escherichia coli* O157:H7 has been widely known as a common cause of acute renal failure in children. There are only a few reports of sporadic Shiga toxin-producing *Escherichia coli*-hemolytic uremic syndrome in adults in the USA. Analyses from the 2011 outbreak of hemolytic uremic syndrome associated with *Escherichia coli* O104:H4 reported that mortality rates are highest in those patients with age >60-years old. Therefore, recognizing Shiga toxin-producing *Escherichia coli*-hemolytic uremic syndrome in older people can help early introduction of the appropriate therapy.

**Case presentation:**

We describe an 86-year-old Caucasian woman, initially treated as suspected thrombotic thrombocytopenic purpura, with worsening neurological and renal functions despite plasmapheresis (plasma exchange). A subsequent normal ADAMTS13 activity level and positive stool sample for *Escherichia coli* O157:H7 confirmed the diagnosis of Shiga toxin-associated hemolytic uremic syndrome. We shifted our management towards aggressive supportive care. Despite conventional treatment, hemolytic uremic syndrome unfortunately led to her death.

**Conclusions:**

Our case demonstrates the importance of recognizing Shiga toxin-producing *Escherichia coli*-hemolytic uremic syndrome as an etiology of microangiopathic hemolytic anemia in older people. According to the current literature, supportive care is the best approach for Shiga toxin-producing *Escherichia coli*-hemolytic uremic syndrome. Therapies such as plasma exchange and eculizumab (a complement inhibitor) are not shown to be effective in Shiga toxin-producing *Escherichia coli*-hemolytic uremic syndrome. There is a dire need to continue research to find better treatment options in this disease entity with a high mortality, particularly in older people.

## Background

Microangiopathic hemolytic anemia (MAHA) results in erythrocyte fragmentation, elevated lactate dehydrogenase (LDH) levels, and microvascular occlusion. MAHA with normal ADAMTS13 activity level consists of a spectrum of disorders that includes hemolytic uremic syndrome (HUS) [1]. HUS can be further categorized into two different subsets: classical or typical HUS associated with Shiga toxin-producing *Escherichia coli* (STEC) or invasive pneumococcal infection, and atypical HUS (aHUS) that is not related to STEC and is driven by complement activation and dysregulation.

HUS associated with *E. Coli* O157:H7 has been widely recognized as one of the main causes of acute renal failure in children. Supportive care and dialysis have been previously known as the mainstream therapy in STEC-HUS in children. However, there are not many cases of STEC-HUS reported in adults worldwide. The report of an outbreak of HUS associated with STEC O104:H4 in Germany in 2011 showed the highest rate of mortality in adults >60-years old [2]. The rarity with which this illness is encountered in adults often delays diagnosis and limits treatment options for this illness.

In this case report, we demonstrate a case of an 86-year-old woman affected by STEC-HUS which is an illness more common in children. Recognizing HUS in older people and differentiating it from other MAHA entities can help early diagnosis and treatment.

## Case presentation

An 86-year-old Caucasian woman was transferred to our medical intensive care unit from another medical facility to provide a higher level of care for thrombotic microangiopathy and acute renal failure. She presented to an emergency department with an acute onset of abdominal pain and bright red blood per rectum. She reportedly had some breathing difficulty. A computed tomography (CT) of her abdomen had shown nonspecific findings suggestive of colitis and therapy had been started with levofloxacin and metronidazole. Urine studies did not have features of urinary tract infection. She was intubated electively at the other hospital due to altered mental status and need for supplemental oxygen prior to her transfer. Also of note, her family reported that she had some difficulties with her speech; this was in the form of difficulties with word-finding alongside slurring of speech. She had no other neurological deficits at that time. A diagnosis of MAHA with a suspicion for thrombotic thrombocytopenic purpura (TTP) was made and she was transferred to our hospital for a higher level of medical care. On her arrival at our institution, she continued to receive metronidazole, cefepime, and tigecycline.

She had medical comorbidities of essential hypertension, hyperlipidemia, and hypothyroidism. She had no reported history of infectious, autoimmune, or hematologic diseases. On physical examination, remarkable skin findings of purpura and ecchymoses were noted. She was intubated and coarse breath sounds were appreciated on examination.

**Fig. 1:**
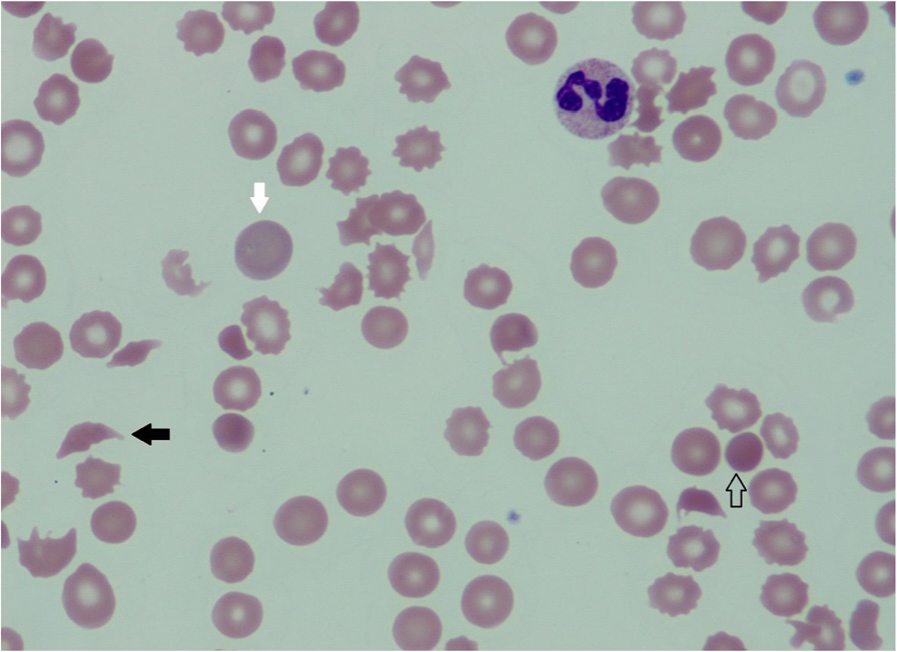
Peripheral blood smear exhibiting features of microangiopathy and marked thrombocytopenia. Severe anemia with schistocytes (*black solid arrow*), reticulocytosis (*white arrow*), and spherocytes (*empty arrow*)

The laboratory data revealed an elevated creatinine, markedly elevated serum LDH, and markedly reduced haptoglobin levels. Her serum ferritin was 739.8 ng/mL, transferrin saturation was 42 %; her cobalamin and folate were within normal limits. A peripheral blood smear (Fig. 1) showed thrombocytopenia and features of MAHA (Table 1).

**Table 1:** Changes in laboratory values during the clinical course

Time	WBC (×10^3^/uL)	Hemoglobin (×10^3^/uL)	Platelet count (×10^3^/uL)	Haptoglobin (mg/dL)	LDH (u/L)	Reticulocyte count	T. Bilirubin (mg/dL)	Creatinine (mg/dL)	BUN (mg/dL)	INR	PTT	Fibrinogen
Initial presentation at another hospital	11	11.1	30	<3.0	2391	3.70 %	2.2	2.49	73	1.20	26.0	577
H0, ICU admission at our center	7.6	6.4	57	NA	4576	6.20 %	1	6.8	112	1.43	35.8	412
H1, 1 day after receiving plasmapheresis	7.4	7.9	62	NA	992	NA	1	5.79	104	1.36	30.9	NA
H2, 2 days after receiving plasmapheresis	14.9	8.2	58	NA	1639	4.90 %	1.5	6.22	122	NA	NA	NA
H3, day stopped plasmapheresis	16.8	8.9	33	NA	2489	4.10 %	2.1	4.78	117	1.40	38.8	148

Timeline of patient’s clinical course and hemolysis laboratory data changes with interventions. H0: hospital day 0; H1: hospital day 1; H2: hospital day 2; H3: hospital day 3, NA: not available. *BUN* blood urea nitrogen, *ICU* intensive care unit, *INR* international normalized ratio, *LDH* lactate dehydrogenase, *PTT* partial thromboplastin time, *T.* total, *WBC* white blood cell

We approached the case as MAHA with a high suspicion for TTP. However, STEC-HUS was also high in our differential diagnosis since she initially had bloody diarrhea. We decided to start plasma exchange (PLEX) alongside methylprednisolone 1 mg/kg twice daily while awaiting ADAMTS13 activity and Shiga-toxin assays. She also underwent hemodialysis for acute renal failure. After an initial improvement in hemolysis and thrombocytopenia, her laboratory and clinical parameters of MAHA failed to improve in subsequent days of hospitalization. Three days later, ADAMTS13 activity level was reported as 92 % (normal range, 68 to 163 % activity); her stool sample was positive for STEC and a culture was positive for *E. coli* O157:H7. These findings were consistent with a diagnosis of STEC-HUS; therefore we recommended discontinuation of corticosteroid and plasmapheresis. We had a lengthy discussion with the family members regarding the limited efficacy of available treatment options. Given her worsening clinical condition, the family decided on palliative measures; she died on her fourth day of hospitalization at our institution.

## Discussion

STEC-HUS is a life-threatening illness that is characterized by MAHA, thrombocytopenia, and acute renal injury which affects 6 to 9 % of STEC infections [3]. Less commonly, neurological involvement is also found to be associated with STEC-HUS infection. The pathophysiology behind STEC causing organ damage is through vasculoendothelial injury. Shiga toxin (Stx) is released by these toxic strains of bacteria into the gastrointestinal tract system and gets absorbed into the systemic circulation where it binds to globotriaosylceramide (Gb3) on the surface of vascular endothelial cells in different target organs, particularly the kidney and brain. Stx injures endothelial cells by inhibiting protein synthesis, inducing broad inflammatory response, and releasing cytokines and chemokines. This endothelial cell injury increases risk of thrombosis and organ damage [4]. Shiga toxin also activates the alternative pathway of complement system by binding to factor H proteins, known as complement control proteins, and reducing their cell surface activity [5]. Furthermore, Stahl *et al*. studied the effects of STEC on complement system and illustrated the increased binding of microparticles from platelets and monocytes in the plasma of patients with HUS to C3 and C9 complement factors, thereby activating them, during the acute phase of the illness causing inflammatory and prothrombotic changes in different organs [6].

The usual presentation of STEC-HUS starts with a prodromal illness with abdominal pain, vomiting, and diarrhea that precedes MAHA, thrombocytopenia, and acute kidney injury (AKI) by 5 to 10 days. MAHA is characterized by hemoglobin levels usually less than 8 g/dL, negative Coombs test, elevated serum indirect bilirubin concentration, elevated serum LDH level, decreased serum haptoglobin concentration and a peripheral blood smear showing a large number of schistocytes and reticulocytes and nucleated red blood cells. Thrombocytopenia is characterized by a platelet count below 150,000/mm^3^. AKI is much harder to define since the severity of renal involvement in STEC-HUS varies from hematuria and proteinuria to development of severe AKI and oliguria requiring dialysis. Studies have shown that about 50 % of patients with HUS require dialysis during the acute phase [5, 7]. There can be additional organ involvements in HUS, with 20 to 25 % of patients with HUS developing life-threatening neurological involvement with severe neurological symptoms such as lethargy, apnea, coma, seizures, cortical blindness, and hemiparesis [3]. Brain magnetic resonance imaging in patients with severe neurological involvement revealed abnormal findings in the basal ganglia, thalami, and brainstem [8]. Severe central nervous system (CNS) involvement has been shown to be associated with higher mortality [4].

Although we are more familiar with the occurrence of the disease in children, there have now been a few studies recognizing the occurrence of STEC-HUS in adults. Gould *et al*. analyzed data from Foodborne Diseases Active Surveillance Network (FoodNet) population-based surveillance for *E. Coli* O157:H7 infection and for HUS from the years 2000 to 2006 in eight different states in the USA to further explore demographic risk factors for the development of STEC-HUS and to evaluate mortality rates associated with STEC-HUS by age. From their data analyses, they reported that young females of <5-years old had the highest risk for developing HUS following *E. Coli* O157:H7 infection, and people >60-years old had the highest mortality rate associated with *E. Coli* O157:H7 infection with or without HUS [9]. In the 2011 outbreak of HUS associated with *E. Coli* O104:H4 in Germany, the frequency of neurological symptoms was higher in adults than in children which led to higher mortality rates. It was found that deaths occurred acutely during initial hospitalization mainly due to neurological damage with symptoms consistent with cerebral edema and infarction, sepsis, and electrolyte disturbances [2].

In addition to basic supportive care, multiple treatment modalities ranging from antithrombotic agents, PLEX, tissue-type plasminogen activator, oral Stx-binding agent, and eculizumab have been studied in the management of STEC-HUS. We will mainly focus on PLEX and eculizumab therapy in this discussion as they are the two most frequently studied therapies in HUS. PLEX has been attempted as treatment for STEC-HUS. The rationale behind PLEX use in STEC-HUS would be to remove Shiga-like toxin and prothrombotic factors caused by the toxin and inflammatory mediators, and replace them with coagulation, tissue and complement factors [6]. According to the 2010 guidelines of the American Society for Apheresis, PLEX is listed as one of the recommended treatments for STEC-HUS [10]. However, analysis of the 2011 *E. Coli* O104:H4-associated HUS outbreak in Germany proposed that general supportive care has the same clinical outcome as PLEX in patients of all ages [2]. In our case presentation, we learned that PLEX led to an initial improvement in hemolysis and consumptive thrombocytopenia. PLEX should be initiated early when MAHA is suspected as it is often difficult to differentiate HUS from TTP and there is a time lag to obtain the results for ADAMTS13 activity assay and stool studies [1].

Another therapy that has been studied in STEC-HUS in children is eculizumab, a monoclonal antibody to C5 complement factor blocking complement activation. Eculizumab has been shown to be an effective drug in treatment of complement-mediated HUS (aHUS). There have been case series which have demonstrated the benefit of using eculizumab in children with STEC-HUS and CNS involvement [11, 12]. On the contrary, other studies reported that there was no benefit seen with use of eculizumab in addition to standard medical care in patients (both adults and children) affected in the 2011 outbreak of *E. Coli* O104:H4 [2, 4]. There is, however, a lack of data supporting the use of eculizumab in the older population with STEC-HUS. Further randomized controlled trials are needed to identify whether, when, and in which STEC-HUS cases eculizumab administration is beneficial. The most effective treatment in terms of cost and safety was found to be general supportive care with volume resuscitation, renal replacement therapy, parenteral nutrition, and transfusion of blood products. Despite these therapeutic measures, STEC-HUS is associated with a high risk of mortality especially in older patients [2, 13].

## Conclusions

This case demonstrates the importance of recognizing STEC-HUS as an etiology of MAHA in older people. Currently, literature data support the view that supportive care is the best approach for STEC-HUS. Conventional therapies for other MAHA, such as PLEX and eculizumab, are not shown to be effective in STEC-HUS. There is a dire need to continue research and randomized controlled trials to find better treatment options for STEC-HUS.
